# Cortical astrocytes regulate ethanol consumption and intoxication in mice

**DOI:** 10.1038/s41386-020-0721-0

**Published:** 2020-05-28

**Authors:** E. K. Erickson, A. J. DaCosta, S. C. Mason, Y. A. Blednov, R. D. Mayfield, R. A. Harris

**Affiliations:** 1 Waggoner Center for Alcohol and Addiction Research, The University of Texas at Austin, Austin, TX 78712-01095 USA; 2 Institute for Cell and Molecular Biology, The University of Texas at Austin, Austin, TX 78712-0195 USA; 3 Department of Neuroscience, The University of Texas at Austin, Austin, TX 78712-01095 USA

**Keywords:** Astrocyte, Addiction

## Abstract

Astrocytes are fundamental building blocks of the central nervous system. Their dysfunction has been implicated in many psychiatric disorders, including alcohol use disorder, yet our understanding of their functional role in ethanol intoxication and consumption is very limited. Astrocytes regulate behavior through multiple intracellular signaling pathways, including G-protein coupled-receptor (GPCR)-mediated calcium signals. To test the hypothesis that GPCR-induced calcium signaling is also involved in the behavioral effects of ethanol, we expressed astrocyte-specific excitatory DREADDs in the prefrontal cortex (PFC) of mice. Activating G_q_-GPCR signaling in PFC astrocytes increased drinking in ethanol-naïve mice, but not in mice with a history of ethanol drinking. In contrast, reducing calcium signaling with an astrocyte-specific calcium extruder reduced ethanol intake. Cortical astrocyte calcium signaling also altered the acute stimulatory and sedative-hypnotic effects of ethanol. Astrocyte-specific G_q_-DREADD activation increased both the locomotor-activating effects of low dose ethanol and the sedative-hypnotic effects of a high dose, while reduced astrocyte calcium signaling diminished sensitivity to the hypnotic effects. In addition, we found that adenosine A1 receptors were required for astrocyte calcium activation to increase ethanol sedation. These results support integral roles for PFC astrocytes in the behavioral actions of ethanol that are due, at least in part, to adenosine receptor activation.

## Introduction

Astrocytes contribute to information processing in the central nervous system [1]. Ongoing brain activity activates astrocytic G_q_-GPCR receptors, initiating phospholipase C (PLC)/inositol trisphosphate (IP3)-dependent intracellular calcium elevations, leading to gliotransmitter release and neuromodulation [2]. This recently appreciated mechanism of astrocyte function challenges the long-held view that animal behavior is exclusively mediated by neurons. As such, behavioral pathologies integral to substance use disorders may be driven, at least in part, by changes in astrocyte calcium signaling.

In vitro, ethanol elicits calcium elevations in astrocytes [3–7] and alters astrocyte calcium responses to neurotransmitters [8–10]. Transcriptome sequencing of astrocytes isolated from prefrontal cortex (PFC) of chronic ethanol-exposed mice revealed decreased gene expression related to GPCR signal transduction and calcium [11, 12], suggesting ethanol consumption modulates cortical astrocyte signaling in vivo. Perturbations in astrocyte calcium signaling can have widespread consequences, affecting neuromodulatory processes that regulate a variety of behaviors. This has been elegantly shown in numerous studies that used astrocyte-specific chemogenetic techniques to demonstrate functional roles for astrocyte G_q_-GPCR signaling in anxiety [13], memory [14], cognitive flexibility [15] and food consumption [16, 17].

Studies exploring the role of astrocytes in addiction have focused mainly on the effects of striatal astrocytes in relapse-related behaviors [18–22]. The PFC, however, is a central regulator of ethanol drinking by modulating sensitivity to ethanol’s interoceptive effects [23, 24], attributing motivational salience to ethanol, and regulating the transition from moderate to escalated consumption [25, 26]. Cortical astrocytes are particularly vulnerable to ethanol-induced morphological and transcriptomic changes [11, 27–32], and a recent study shows that expression of a protease in *Drosophila* cortical glia, homologous to mammalian cortical astrocytes, controls ethanol’s acute sedative effects [33]. These studies indicate that cortical astrocytes regulate both molecular and behavioral responses to ethanol.

Here, we manipulated astrocyte calcium signaling in vivo to study the functional role of mammalian cortical astrocytes in ethanol consumption and intoxication. Using astrocyte-specific viral techniques, we demonstrated that activation of cortical astrocyte G_q_-GPCR signaling increases ethanol consumption, while reduced astrocyte calcium signaling decreases ethanol consumption. Acute stimulatory and sedative-hypnotic ethanol-induced phenotypes were also regulated by cortical astrocyte calcium signaling. In addition, we show that adenosine receptors are critical for behavioral regulation by activated astrocytes. Our findings reveal a specific role for PFC astrocytes in acute ethanol-induced behaviors and the escalation of ethanol consumption.

## Materials and methods

### Animals

Adult (7–8 weeks old) male C57BL6/J mice purchased from Jackson Laboratories (Bar Harbor, ME) were used for all experiments. Mice were housed in the Animal Resource Center at The University of Texas at Austin and kept on a standard laboratory diet and water *ad libitum*. For drinking experiments, mice were kept in a reverse 12 h light/dark cycle room. All other experiments were performed under standard 12 h light/dark cycles. All experiments were approved by The University of Texas at Austin Institute for Animal Care and Use Committee and conducted in accordance with NIH guidelines regarding use of animals in research.

### Viruses

Adeno-associated viruses (AAV) under the control of the 681 bp GFAP promoter gfaABC1D and an AAV2/5 serotype were used for astrocyte-specific expression. pAAV-GFAP-hM3D(G_q_)-mCherry (Addgene #50478) was sent to the UNC Viral Vector Core (Chapel Hill, NC) for custom AAV5 production. AAV5-GFAP-GFP was directly purchased from UNC. AAV5-GFAP-tdTomato was provided by Dr. Baljit Khakh (UCLA) (Addgene viral prep #44332-AAV5). AAV5-GFAP-hPMCA2w/b-mCherry (AAV-CalEx) was custom-made at the University of Pennsylvania Vector Core (Philadelphia, PA) using the plasmid pAAV-GFAP-hPMCA2w/b, which was generously provided by Dr. Khakh.

### Stereotaxic surgery

The following coordinates were used to inject 50 nl AAV bilaterally into mouse prefrontal cortex (PFC): 2.1 AP, +/−0.4 ML, 1.4 DV. Injections were given using a Nanoject II (Drummond Scientific, Broomall, PA) at a rate of 23 nl/sec. Two minutes were allowed for viral diffusion after each injection. Mice recovered for three weeks before any biochemical or behavioral testing began.

### Chemicals

The hM3Dq agonist Clozapine-N-Oxide (CNO; HB6149, Hello Bio, Princeton, NJ) was generously provided by Dr. Thomas Kash (UNC) for G_q_-DREADD activation experiments. CNO was dissolved in sterile saline for i.p. injections. Injectable ethanol (100% stock; Aaper Alcohol and Chemical, Shelbyville, KY) solutions were prepared in 0.9% saline (20%, v/v). Dipropylcyclopentyl xanthine (DPCPX) was obtained from Millipore Sigma (Burlington, MA) and prepared in 0.9% saline and 1% Tween-80. Gaboxadol (Sigma-Aldrich, St. Louis, MO) was dissolved in 0.9% saline. Solutions were prepared fresh immediately before use on each experimental day.

### Immunohistochemistry

AAV-hM3Dq mice were given i.p. injections of saline or CNO (1 mg/kg) 90 min prior to being anaesthetized with isoflurane. AAV-CalEx and AAV-tdTomato mice were administered i.p. injections of saline or ethanol (1.25 g/kg) 90 min prior to anesthetization. Mice were given transcardial perfusions of 4% paraformaldehyde (PFA) before brains were removed and post-fixed in PFA. Brains were frozen in Optimal Cutting Temperature (Tissue-Tek OCT; Sakura Finetek, Torrance, CA) and sectioned into 30 μm slices. CNO-induced astrocyte activation was assessed by immunostaining using primary antibodies for c-Fos and glutamine synthetase (GS) (Millipore) [14, 15]. Cell specificity of viral transduction was assessed with mCherry (Sicgen), astrocyte (GS), neuronal (NeuN, Millipore), and microglial (IBA1, Wako) staining (Supplemental Figs. 1 and 2). Bilateral images of the PFC (Bregma +2.8 to +2.2) were acquired using a Zeiss Axiovert 200 M fluorescent light microscope. Image analysis was performed using ImageJ (version 1.50i). Two 1000 μm^2^ regions of interest (ROI) corresponding to medial PFC were defined for each section. Cell counts were averaged between two to three sections per animal. For c-Fos quantification, a threshold was applied to ROIs and c-Fos was counted using ImageJ’s “Analyze Particles” function. Thresholded c-Fos+ cells colocalizing with GS + cells were counted, along with total GS + cells in each ROI. Percentage of c-Fos+ astrocytes were calculated by dividing colocalized counts by total astrocytes (GS + cells). Cell-type specificity of AAVs were quantified by counting total number of neurons, astrocytes, or microglia in a viral transduced ROI as well as the number of cells of each cell type colocalizing with mCherry. The percentage of each cell type co-expressing mCherry was calculated by dividing colocalized counts by total number of cells.

### Ethanol drinking

Intermittent every-other-day (EOD) access to ethanol increases voluntary drinking in rodents [34]. Mice underwent EOD drinking as described previously [11, 35]. Mice had access to 15% (v/v) ethanol EOD and water every day. Bottle positions were changed daily to control for position preferences. Each point in the graphs represents the average of 2 drinking days with different bottle positions. To control for evaporation and spillage, two bottles containing ethanol and water in an empty cage were also measured daily. Consumption (g/kg body weight/time) and preference (amount of ethanol consumed divided by total fluid consumed per drinking session; a value of >0.5 or 50% indicates preference for ethanol) were calculated for each mouse. For G_q_-DREADD experiments, mice were given CNO (1 mg/kg, i.p.) or saline on drinking days, 30 min prior to ethanol access. Ethanol was provided at the onset of the dark cycle, so that drinking would occur at maximal levels of activity.

### Saccharin preference

Saccharin preference was measured according to the procedure described for ethanol drinking. Briefly, mice had access to increasing concentrations of saccharin EOD and water every day. Saccharin concentration began at 0.00165% and was doubled every 2 drinking days until mice in all experimental groups reached maximum preference. For G_q_-DREADD experiments, mice were given i.p. injections of 1 mg/kg CNO or saline on drinking days, 30 min prior to saccharin access.

### Locomotor activity

To assess stimulatory effects of ethanol, general locomotor activity in response to ethanol treatment was recorded in activity chambers [36]. AAV-G_q_-DREADD mice were given 1 mg/kg CNO or saline (i.p.) 30 min before receiving ethanol or saline. Mice received 1 g/kg or 1.25 g/kg ethanol (i.p.). Five min later, mice were placed in a light- and sound-attenuating ventilated chamber (Med Associates, St. Albans, VT). Infrared light sources and photodetectors were used to measure general activity of each mouse for 30 min [37]. Total activity counts were computer recorded.

### Loss of righting reflex

Loss of righting reflex (LORR) was evaluated to measure the acute sedative-hypnotic effects of ethanol, as described previously [38]. The amount of time it takes for each mouse to become ataxic (unable to right themselves) after an injection of ethanol (3.6 g/kg, i.p.) is recorded as the latency to LORR. When ataxic, mice are placed in the supine position in V-shaped plastic troughs until they can right themselves 3 times within 30 sec. Duration of LORR is calculated as the time from being placed in the supine position until mice regain their righting reflex. We also assessed LORR induced by another GABAergic sedative drug, gaboxadol (55 mg/kg) [38]. For G_q_-DREADD experiments, AAV-G_q_-DREADD mice were given 1 mg/kg CNO or saline (i.p.) 30 min before receiving ethanol. When testing the role of A1 adenosine receptor signaling, 2.5 mg/kg DPCPX or saline pretreatment was given 5 min before the CNO or saline treatment [39].

### Statistical analysis

Prism 8.0 (GraphPad Software, Inc., La Jolla, CA) was used to perform all statistical tests. Graphs were created in Prism 8.0. Data are reported as mean ± S.E.M. values. In some cases, the SEM bars are smaller than the symbols representing the mean. Comparisons involving more than two groups or conditions were calculated using one-way or two-way repeated-measures ANOVA followed by Bonferroni post-hoc tests, while datasets with two groups were analyzed using unpaired Student’s two-tailed *t* tests.

## Results

### Activating astrocyte calcium signaling increases ethanol consumption in ethanol-naïve mice

**Fig. 1:**
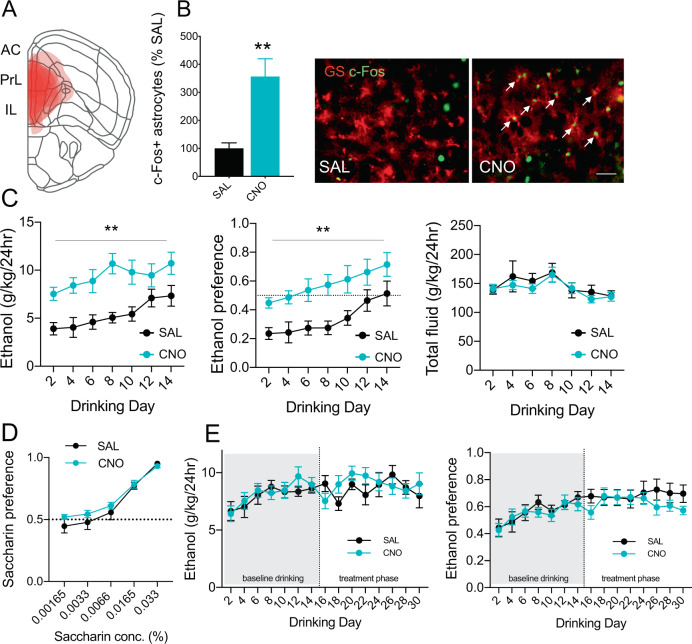
Activating cortical astrocytes increases escalation of ethanol consumption. **a** Diagram of AAV-hM3Dq spread throughout prefrontal cortex. Each layer demonstrates the extent of viral spread of a separate mouse, superimposed over reference coronal slice at +2.00 mm from Bregma (*n* = 12). **b** Quantification of c-Fos+ astrocytes and representative immunohistochemistry images showing c-Fos expression in astrocytes in saline vs. CNO-treated mice expressing AAV-hM3Dq. Colocalized c-Fos and GS indicated by white arrows. Scale bar, 50 μm. ** Students *t* test, *p* = 0.0048 (*n* = 5). **c** AAV-G_q_-DREADD mice treated with CNO (blue lines) exhibit increased ethanol consumption and preference compared with saline-treated mice (black lines). CNO administration does not alter 24-h total fluid. Data were analyzed by two-way repeated-measures ANOVA. **p < 0.01 compared with control (*n* = 6 per group). **d** Saccharin preference for AAV-G_q_-DREADD mice treated with CNO or saline (*n* = 9–10 per group). **e** Ethanol consumption and preference for AAV-G_q_-DREADD mice after escalations in drinking stabilized prior to saline or CNO treatment (*n* = 8–11 per group). Values are mean ± SEM.

We used an AAV expressing hM3Dq conjugated to mCherry under the control of a human GFAP promoter to express excitatory DREADDs in mouse cortical astrocytes. Immunohistochemistry results indicated G_q_-DREADD expression was found in astrocytes, as the mCherry stain was colocalized with the astrocyte marker GS but did not overlap with the neuronal marker NeuN (Supplementary Fig. 1). Viral expression was primarily restricted to prelimbic and infralimbic regions of the medial PFC (Fig. 1a). The DREADD agonist CNO (1 mg/kg) activated astrocyte calcium signaling as indicated by increased c-Fos immunostaining colocalized with astrocytes (Fig. 1b). G_q_-DREADD activation led to a 3-fold increase in c-Fos+ astrocytes compared with saline-treated mice (*p* < 0.005, t(8) = 3.855, Student’s *t* test), consistent with prior studies elevating calcium signaling in astrocytes with excitatory DREADDs [14, 16, 40]. CNO did not alter c-Fos levels in non-astrocytic cells (data not shown). Previous studies have shown no effect of CNO administration on c-Fos or astrocyte calcium levels in mice not expressing DREADDs, indicating CNO effects are dependent on G_q_-DREADD expression [41, 42].

We next investigated the effect of astrocyte calcium activation on initial escalation of ethanol consumption and preference in ethanol-naïve mice. Ethanol drinking was measured in AAV-G_q_-DREADD mice using an EOD drinking test. Compared with saline treatment, astrocyte-specific calcium activation (1 mg/kg CNO, i.p., 30 min before ethanol exposure on drinking days) robustly increased both ethanol consumption and preference (two-way ANOVA, main effect of treatment on ethanol consumption: F_1,10_ = 14.63, *p* < 0.005, main effect of treatment on ethanol preference: F_1,10_ = 10.32, *p* < 0.01; Fig. 1c). Total fluid intake did not differ between CNO- and saline-treated groups (Fig. 1c). We also examined EOD drinking in mice expressing a control virus (GFP) to determine possible off-target effects of CNO. However, CNO did not alter ethanol consumption or preference compared with saline in non-DREADD-expressing mice (Supplementary Fig. 3A). We then measured preference for saccharin to determine whether altered sweet taste perception could account for the increased ethanol consumption found in mice with astrocyte G_q_-DREADD activation. CNO (1 mg/kg) did not change EOD saccharin intake or preference in G_q_-DREADD-expressing mice (Fig. 1d).

Next, we examined the effect of increased astrocyte calcium signaling in ethanol-exposed G_q_-DREADD-expressing mice using the EOD procedure. AAV-G_q_-DREADD mice established stable baseline levels of ethanol consumption and preference for 28 days (14 drinking days) in an EOD drinking test before treatment with CNO (1 mg/kg) or saline began (Fig. 1e). Astrocyte calcium activation by CNO over the next 16 drinking days did not affect maintenance of ethanol consumption or preference in these mice (Fig. 1e). Our results suggest astrocyte calcium signaling alters the initial escalation of ethanol drinking, thus increasing drinking behavior in ethanol-naïve mice but not in mice with prior ethanol experience.

### Reducing cortical astrocyte calcium signaling suppresses escalation of ethanol drinking in ethanol-naïve mice

**Fig. 2:**
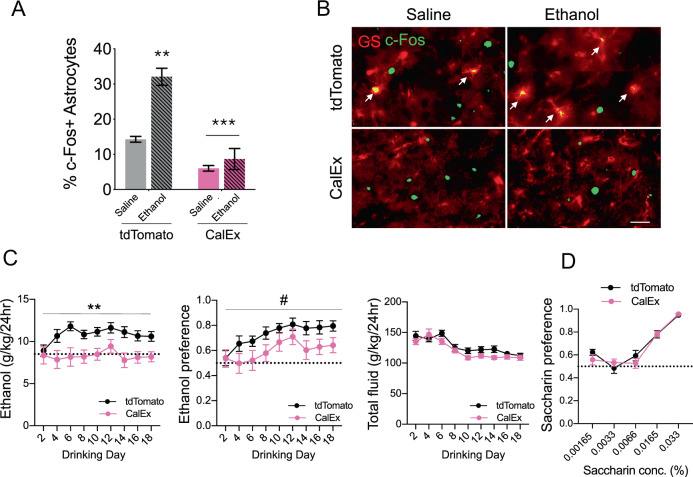
Reducing cortical astrocyte calcium signaling decreases ethanol consumption. **a** Quantification of c-Fos+ astrocytes in AAV-tdTomato (gray) or AAV-CalEx (pink) expressing mice treated with saline (solid bars) or 1.25 g/kg ethanol (hatched bars). Data were analyzed by two-way ANOVA followed by Bonferroni’s multiple comparisons test. **p < 0.005 compared with saline control, ***p < 0.0005 (main effect of virus). **b** Representative immunohistochemistry images of c-Fos colocalization with an astrocyte marker (GS) in tdTomato or CalEx-expressing mice treated with saline or ethanol. Scale bar, 50 μm. **c** Ethanol consumption, preference, and total fluid intake in mice expressing a control virus (black line) or AAV-CalEx (pink line). In the ethanol intake panel, the dotted line represents the amount of ethanol consumed on the first full day of drinking averaged for both groups (8.5 g/kg). In the panel showing ethanol preference, the dotted line indicates a value of 0.5, or no preference (*n* = 9–10 per group). Data were analyzed by two-way repeated-measures ANOVA, **p < 0.01, ^#^*p* < 0.1 compared with control. **d** Saccharin preference in mice expressing a control virus (black line) or AAV-CalEx (pink line) (*n* = 6 per group). Values are mean ± SEM.

We next assessed whether cortical astrocyte calcium signaling is necessary to increase ethanol consumption in an EOD drinking experiment. To inhibit astrocyte calcium signaling, we expressed astrocyte-specific AAV-CalEx [43] in mouse PFC. AAV-CalEx encodes hPMCAw/b, a plasma membrane ATPase that transports calcium out of the cell, which reduces basal calcium levels, amplitude and duration of spontaneous calcium signals, as well as pharmacologically evoked calcium elevations in astrocytes [43]. We confirmed astrocyte-specific expression of AAV-CalEx, observing colocalization of mCherry with GS but not IBA1 or NeuN+ cells (Supplementary Fig. 2). Astrocytes expressing CalEx exhibit generally normal physiological characteristics [43] and AAV-CalEx mice showed no differences in the amount of GS + cells in the PFC compared with control virus-expressing mice (Supplementary Fig. 2) We then assessed baseline and ethanol (1.25 g/kg, i.p.)-induced astrocyte-specific c-Fos expression in AAV-CalEx and control virus-expressing mice. We observed less astrocytic c-Fos in CalEx mice compared with the control group, indicating reduced baseline astrocyte calcium signaling (Fig. 2). A two-way ANOVA revealed a main effect of virus (F_1,6_ = 61.6, *p* < 0.0005), ethanol treatment (F_1,6_ = 25.6, *p* < 0.005) and a virus x ethanol interaction (F_1,6_ = 14.1, *p* < 0.01) on astrocyte c-Fos expression. Ethanol elevated astrocytic c-Fos expression in control virus-expressing mice (*p* < 0.005), but not in AAV-CalEx-expressing mice (Bonferroni’s multiple comparisons test, Fig. 2).

In an EOD drinking test, mice expressing AAV-CalEx drank significantly less ethanol than their control (AAV-tdTomato) counterparts (two-way ANOVA, main effect of virus: F_1,17_ = 9.20, *p* < 0.01, Fig. 2c). Ethanol intake during the first two drinking days was similar between the groups, however, over time the control mice escalated their consumption, while AAV-CalEx mice did not. Preference increased in both groups, yet control mice consistently showed higher preference than AAV-CalEx mice, although this difference was not statistically significant (two-way ANOVA, main effect of virus: F_1,17_ = 3.36, *p* = 0.08; Fig. 2c). No difference in total fluid intake was observed between groups (Fig. 2c). In a separate experiment, we studied preference for sweet taste in control and AAV-CalEx mice but found no group differences in saccharin preference (Fig. 2d).

### Astrocytes regulate locomotor response to ethanol

**Fig. 3:**
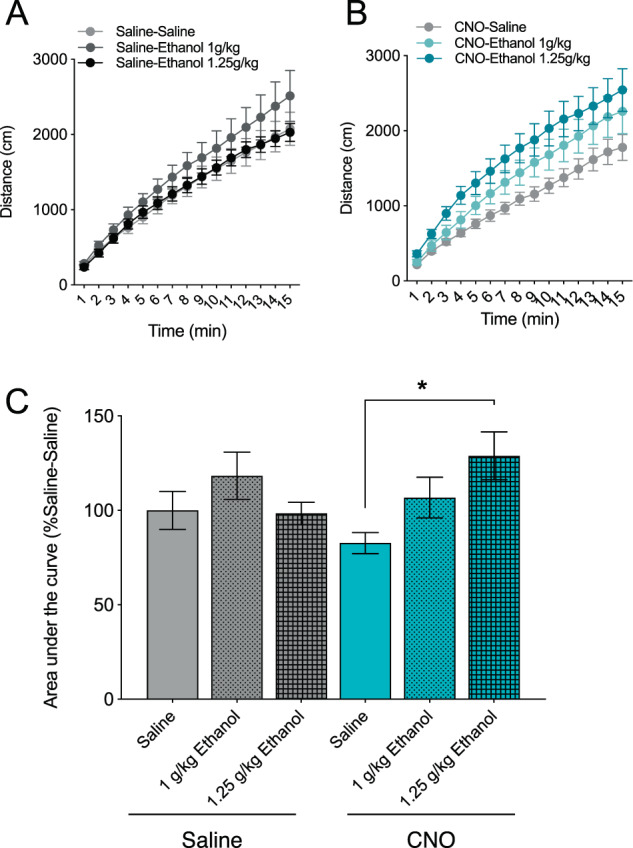
Astrocytes regulate locomotor response to ethanol. Cumulative distance traveled over a 15-minute period in (**a**) saline- or (**b**) CNO-pretreated AAV-G_q_-DREADD-expressing mice injected with saline (SAL) or ethanol (1 g/kg or 1.25 g/kg) (*n* = 10–12 per group). **c** distance traveled represented by area under the curve for each treatment group, expressed as a percentage based on the saline-saline group. Data analyzed by two-way ANOVA with Bonferroni’s multiple comparisons test, *p < 0.05. Values are mean ± SEM.

Ethanol’s locomotor effects have been associated with propensity for high or low ethanol consumption in mice [44]. We examined the effect of astrocyte activation on ethanol-induced locomotor activity using two low doses of ethanol (1, 1.25 g/kg). Two-way ANOVA revealed a significant ethanol x CNO interaction (F_2,62_ = 3.17, *p* < 0.05). Compared with control mice, ethanol treatment did not significantly alter horizontal locomotor activity (shown as the distance traveled over 15 min and as the respective area under each curve) in saline-pretreated AAV-G_q_-DREADD mice (Fig. 3a). However, mice pretreated with CNO (1 mg/kg) showed changes in locomotor activity in response to ethanol (Fig. 3b). Treatment with 1.25 g/kg ethanol significantly increased horizontal activity compared within CNO-pretreated mice (*p* < 0.05) but not saline-pretreated mice (Fig. 3c). Pretreatment with CNO alone did not alter baseline locomotor activity in AAV-G_q_-DREADD mice (Supplementary Fig. 4). In a separate experiment, we investigated the potential role of astrocyte calcium signaling on motor incoordination induced by administration of 2 g/kg ethanol. Compared with saline pretreatment, CNO did not alter ethanol-induced motor incoordination in mice expressing AAV-G_q_-DREADD (Supplementary Fig. 5). Taken together, these results suggest that cortical astrocyte calcium signaling regulates the acute stimulatory effects of low dose ethanol.

### Astrocytes regulate sedative-hypnotic effects of ethanol

**Fig. 4:**
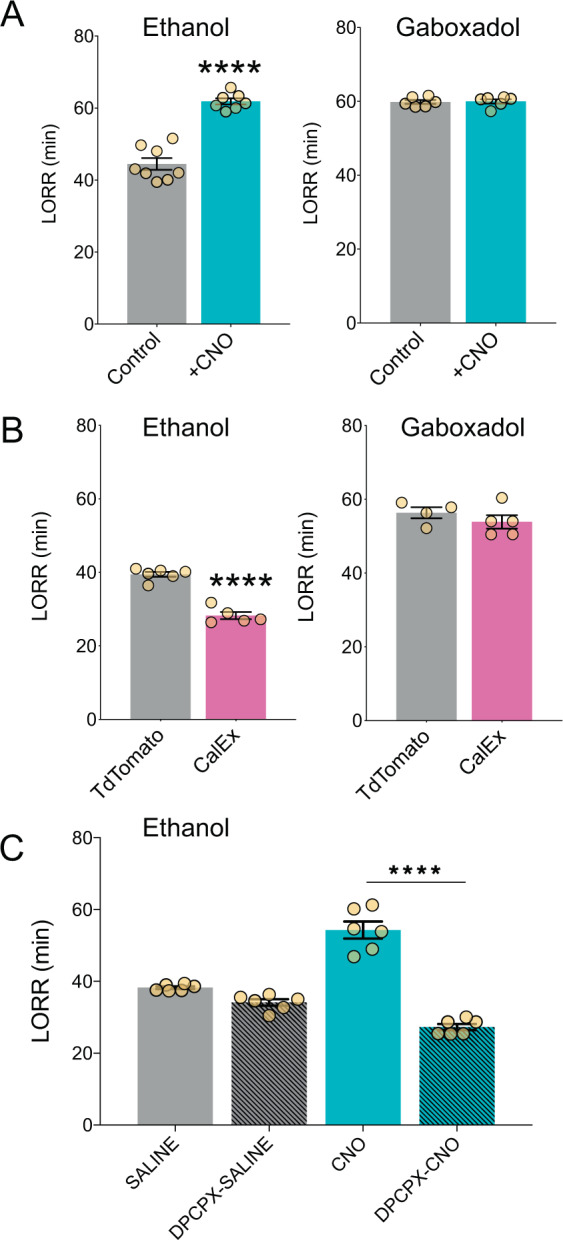
Astrocyte calcium signaling mediates duration of ethanol-induced hypnosis. **a** Loss of righting reflex (LORR) duration in CNO- vs saline-pretreated AAV-DREADD-expressing mice and **b** AAV-CalEx vs AAV-tdTomato (control) expressing mice. Data analyzed by Student’s *t* test. **c** Effect of DPCPX (2.5 mg/kg) pretreatment on duration of ethanol-induced LORR in saline- or CNO-treated AAV-G_q_-DREADD-expressing mice. Data analyzed by two-way ANOVA with Bonferroni’s multiple comparisons test, ****p < 0.0001. Values are mean ± SEM.

Ethanol consumption may also be associated with sensitivity to the hypnotic effects of ethanol, as measured in rodents by duration of the loss of righting reflex (LORR) [45]. Therefore, we examined the effect of astrocyte-specific G_q_-DREADD activation on ethanol (3.6 g/kg)-induced LORR. CNO (1 mg/kg) increased LORR duration by nearly 20 min in AAV-G_q_-DREADD mice (*p* < 0.0001, t(13) = 9.018, Student’s *t* test, Fig. 4a), but not in mice not expressing DREADDs (Supplementary Fig. 3B). Duration of LORR induced by administration of another sedative, gaboxadol (55 mg/kg), was not altered by astrocyte G_q_-DREADD activation (Fig. 4a), indicating that astrocyte calcium signaling selectively increases the sedative-hypnotic effects of ethanol.

We next examined the effect of reduced astrocyte calcium signaling on ethanol-induced LORR. Mice expressing AAV-CalEx in PFC astrocytes showed reduced duration of ethanol-induced LORR compared with mice expressing a control virus (*p* < 0.0001, t(9) = 9.743, Student’s *t* test; Fig. 4b). No change in duration was observed in response to gaboxadol-induced LORR (Fig. 4b).

### Adenosine receptors are required for astrocyte-enhanced ethanol sedation

Activation of G_q_-GPCR pathways in astrocytes can release gliotransmitters such as ATP, which is typically rapidly degraded into adenosine [13, 46, 47]. Astrocyte-derived adenosine has been implicated in several behavioral domains including fear, feeding, depression, and sleep [13, 16, 48, 49]. In the PFC, inhibitory adenosine A1 receptors are the most abundant subtype [11]. A1 receptors in this region are specifically known to regulate wakefulness after anesthesia [50]. We hypothesized that AAV-G_q_-DREADD activation increases extracellular adenosine and activation of neuronal adenosine A1 receptors, contributing to increased duration of ethanol-induced LORR. We used an A1 antagonist, DPCPX, to block activation of A1 receptors in AAV-G_q_-DREADD mice [39]. In the absence of astrocyte G_q_-DREADD activation, DPCPX (2.5 mg/kg) pretreatment did not alter ethanol-induced sedation. However, DPCPX completely abolished the increased sedative effects of ethanol produced by astrocyte G_q_-DREADD activation (Fig. 4c). Thus, adenosine A1 receptors are necessary for astrocyte G_q_-GPCR activation to increase ethanol’s sedative-hypnotic effects.

## Discussion

Astrocytes have a growing reputation as pivotal behavioral regulators [2, 51, 52], but the contribution of astrocytes to addiction is still largely undefined. Here, we delineate novel roles for PFC astrocyte calcium signaling in specific behavioral responses to ethanol.

Activating cortical astrocytes increased ethanol drinking in ethanol-naïve mice, but not in mice with a history of escalated drinking. In contrast, reducing astrocyte calcium with AAV-CalEx prevented normal increases in ethanol intake, indicating a requirement for intact cortical astrocyte calcium signaling to induce escalations in drinking. This suggests that astrocytes regulate initial sensitivity to ethanol, and pairing ethanol exposure with astrocyte activation may produce synergistic effects. Indeed, we found that cortical astrocyte calcium signaling enhanced acute behavioral responses to ethanol (e.g., increased sensitivity to locomotor-activating effects of low doses and sedative-hypnotic effects of an anesthetic dose). Suppressed astrocyte calcium, however, led to decreased sensitivity to ethanol’s sedative effects, which suggests that cortical astrocyte calcium signaling is a key driver of certain behavioral indicators of ethanol intoxication.

The PFC is functionally linked to a vast array of subcortical structures which regulate behavioral state transitions that coordinate locomotor activity and sedation [26]. For instance, noradrenergic inputs projecting from the locus coeruleus directly to the PFC are finely tuned to regulate sleep-to-wake transitions and general locomotor activity [53]. Indeed, this pathway is a critical mediator of cortical arousal, promoting emergence from ethanol-induced coma [54–56]. Noradrenaline levels in the PFC also correlate with hyperactivity following administration of stimulant drugs [57]. Astrocytes could be influencing transitions in cortical activity underlying arousal [58]. Cortical astrocytes appear to be a primary target of noradrenaline, inducing rapid calcium elevations through stimulation of astrocytic noradrenergic receptors [59–62]. Altered cortical astrocyte calcium is also observed in response to anesthetic drugs, and has been proposed as a non-neuronal mechanism for their sedative action [63, 64]. These changes in astrocyte activity, resulting in gliotransmitter release or adjustments in neurotransmitter uptake, could alter signal processing over thousands of synapses, thus controlling cortical state transitions mediating attention and arousal [64–66]. Consequently, cortical astrocytes may function as mediators of neuromodulatory processes that regulate responses to ethanol. Sensitivity to stimulatory and sedative effects of ethanol, regulated by astrocyte signaling, may influence initial escalation of ethanol consumption.

Over long-term exposure, other cell-types and brain regions could become more influential in regulating ethanol drinking. In mouse and human ethanol-dependent PFC, genes involved in astrocyte function are prominently dysregulated [11, 12, 27, 29, 67]. Generally, genes indicating astrocyte reactivity and inflammation tend to increase, while genes associated with homeostatic functions and synaptic regulation decrease with ethanol exposure [11, 12]. Importantly, ethanol exposure alters astrocyte calcium signaling [3–10], and although chronic effects have not been characterized, transcriptome data suggest that long-term ethanol exposure leads to lasting perturbations in calcium-related signaling pathways in cortical astrocytes [11, 12]. For instance, astrocytes isolated from ethanol-dependent mouse PFC show decreased expression of a network of genes encoding neurotransmitter receptors, synaptic protein interactors, and regulators of downstream processes such as PLC activity, IP3 synthesis and CREB phosphorylation [12]. Activating G_q_-GPCR signaling in PFC astrocytes in mice repeatedly exposed to ethanol may have very different functional consequences than the same manipulation in ethanol-naïve mice. It is also relevant to note that G_q_-GPCR signaling is one of many signaling mechanisms used by astrocytes to modulate brain activity. Different pathways, such as astrocytic G_i_-GPCR signaling could also be involved in initial ethanol responses, or compensate for altered astrocyte function in ethanol-dependent states [68]. Functional studies of astrocyte GPCR and calcium signaling will be necessary to better understand the role of astrocytes in acute and chronic ethanol actions.

**Fig. 5:**
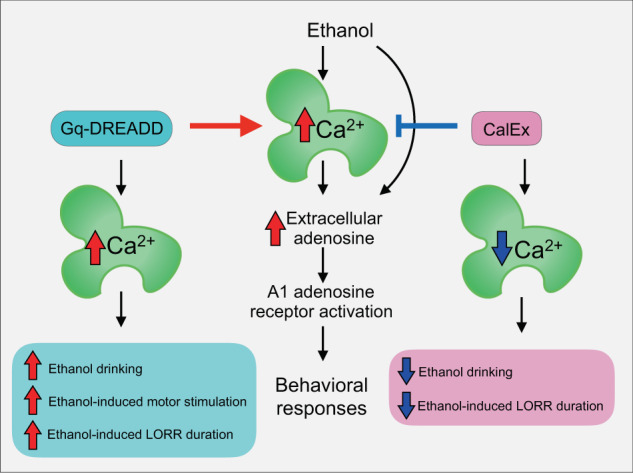
Schematic representing behavioral results and potential adenosine involvement in astrocyte calcium-mediated ethanol drinking and intoxication behaviors. Activating astrocyte calcium with G_q_-DREADD increased ethanol drinking and intoxication phenotypes, while reducing astrocyte calcium with CalEx decreased ethanol drinking and intoxication. A1 adenosine receptor activation was required for the increased sedative effect of astrocyte calcium activation. Thus, some behavioral effects of ethanol may be partly mediated by astrocyte calcium activation and increased adenosine receptor signaling.

Our results suggest that astrocytes regulate ethanol responses by adjustments in purinergic signaling. We found that A1 adenosine receptors were necessary for astrocyte calcium activation to increase ethanol sedation. The dose of DPCPX we used did not affect ethanol-induced LORR duration in control mice, but completely abolished the increased sedation caused by astrocyte DREADD activation. Astrocytes are known to release ATP following GPCR-activated calcium elevations [13, 46], which can lead to increased extracellular adenosine. Ethanol itself induces elevations in adenosine tone [69] that mediate its sedative effects [70–72]. A1 receptor agonists increase ethanol-induced sleep time in mice [73], and A1 receptor antagonism in the basal forebrain attenuates ethanol-induced sleep in rats [74]. More recent work has identified a relationship between ethanol’s subjective sedating effects and cerebral A1AR availability in humans [75]. Based on our preliminary results, we speculate that astrocytes may moderate these adenosine-mediated ethanol effects (Fig. 5). Astrocyte-induced increases in adenosine could also contribute to changes in other ethanol-related phenotypes, including ethanol consumption [76–79]. Antagonism of A1, but not A2A adenosine receptors decreased binge-like ethanol intake in B6 mice [44]. A1 receptors have also been implicated in attenuating withdrawal-related anxiety [80] and enhancing anxiolytic-like effects of ethanol [81].

In addition to adenosine, ethanol interacts with multiple neuromodulator systems in brain that can be regulated by astrocyte calcium activation. Moreover, aside from evoking astrocytic release of a variety of gliotransmitters, intracellular calcium elevations can induce other functional alterations in astrocytes that affect neuronal signaling [2]. Also, the degree of activation and downstream consequences depend on neuronal input and behavioral state [14, 82, 83]. Reductions in astrocyte calcium signaling could also have widespread outcomes on glial and neuronal functioning. For instance, astrocytic CalEx expression in mouse nucleus accumbens enhanced the function of the glial GABA transporter GAT3, reducing the levels of synaptic GABA and altering circuit function [43]. GAT3 plays a critical role in mediating ethanol preference in rats, and its mRNA expression is decreased in human alcohol-dependent brain as well as cortical astrocytes from ethanol-exposed mice [12, 84]. It’s possible that CalEx expression in PFC astrocytes increased GAT3 function, which contributed to decreased ethanol consumption. Whether altered astrocyte calcium signaling in cortex produces functional changes in neurotransmitter uptake that contribute to ethanol consumption and intoxication warrants future investigation.

In summary, we provide evidence that cortical astrocytes modulate behavioral effects of ethanol. We show that ethanol-induced locomotor stimulation, ethanol-induced sedation, and ethanol consumption are all regulated by astrocyte calcium signaling. Thus, astrocytes may influence early behaviors associated with the development of alcohol use disorders.

## Funding and disclosure

This work was supported by NIH/NIAAA INIA Consortium U01 AA025479 to RAH, U01 AA020926 (INIA) to RDM, R01 AA012404 to RAH, and F31 AA025508 to EKE. The authors declare no conflict of interest.

## Supplementary information

Supplemental Data
