# Supplementary material for: Cortical astrocytes regulate ethanol consumption and intoxication in mice
Source: Neuropsychopharmacology. 2020 May 28;46(3):500–8. doi: 10.1038/s41386-020-0721-0 (PMC8027025; doi:10.1038/s41386-020-0721-0)
Supplement: Supplementary file 1 — Supplemental Data [file 41386_2020_721_MOESM1_ESM.pdf]

Cortical astrocytes regulate ethanol consumption and intoxication in mice

E. K. Erickson*[1,2], A. J. DaCosta[1], S. C. Mason[1], Y. A. Blednov[1], R. D. Mayfield[1,3], R. A. Harris[1,3]

* Corresponding Author

[1] Waggoner Center for Alcohol and Addiction Research, The University of Texas at Austin, Austin, TX, 78712-01095, USA

[2] Institute for Cell and Molecular Biology, The University of Texas at Austin, Austin, TX, 78712-0195, USA

[3] Department of Neuroscience, The University of Texas at Austin, Austin, TX, 78712-01095, USA

Supplementary Information

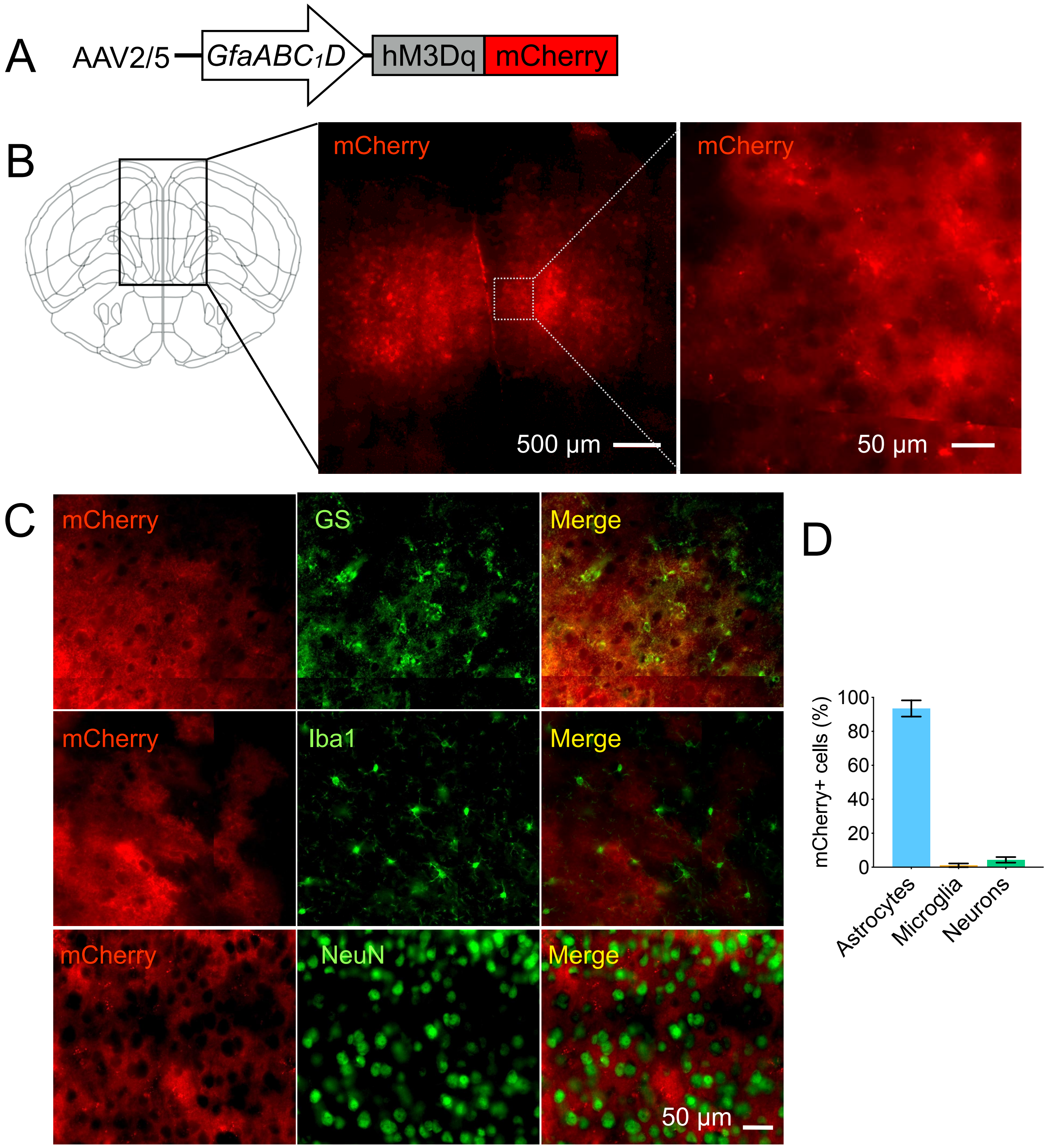

Supplementary Figure 1. Astrocyte-specific AAV schematic, localization and cell-type verification for AAV-G_q-DREADD. (**A**) Schematic of viral construct used to produce AAV-G_q-DREADD. (**B**) Localization and overview of AAV-G_q-DREADD expression in PFC, coronal reference slice at +2.00mm from Bregma. (**C**) Cell-type marker immunohistochemistry showing overlap of AAV-G_q-DREADD (mCherry) with GS+ astrocytes, IBA1+ microglia, and NeuN+ neurons in PFC. (**D**) Quantification of cell-type specificity of AAV-G_q-DREADD expression shows the percentage of mCherry+ cells that colocalized with an astrocytic, microglial, or neuronal marker within the area transduced by the astrocyte-targeted AAV (n = 3, 3 slices per brain). Values are mean ± SEM.

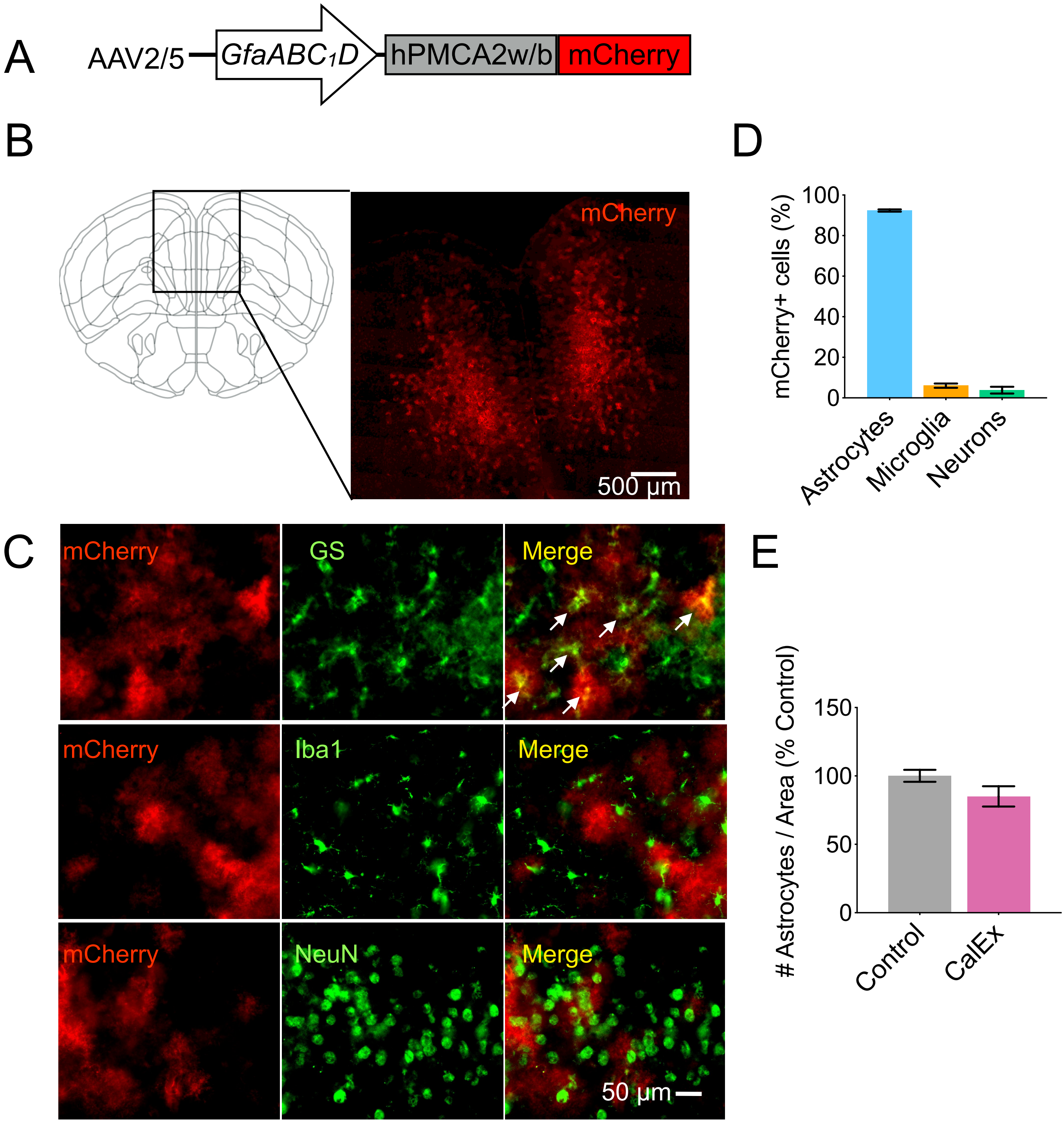

Supplementary Figure 2. AAV schematic and cell-type marker verification for AAV-CalEx. (**A**) Schematic of viral construct used to produce AAV-CalEx. (**B**) Localization and overview of AAV-CalEx expression in PFC, coronal reference slice at +2.00mm from Bregma. (**C**) Overlap of AAV-CalEx (mCherry) with GS+ astrocytes, IBA1+ microglia, and NeuN+ neurons in PFC. (**D**) Quantified cell-type specific expression of AAV-Calex shows the percentage of mCherry+ cells that colocalized with markers for astrocytes, microglia, or neurons (n = 4, 3 slices per brain). (**E**) The number of astrocytes (GS+ cells) in PFC of AAV-CalEx expressing mice was consistent with levels observed in mice expressing a control virus (tdTomato) (n = 4, 3 slices per brain). Values are mean ± SEM.

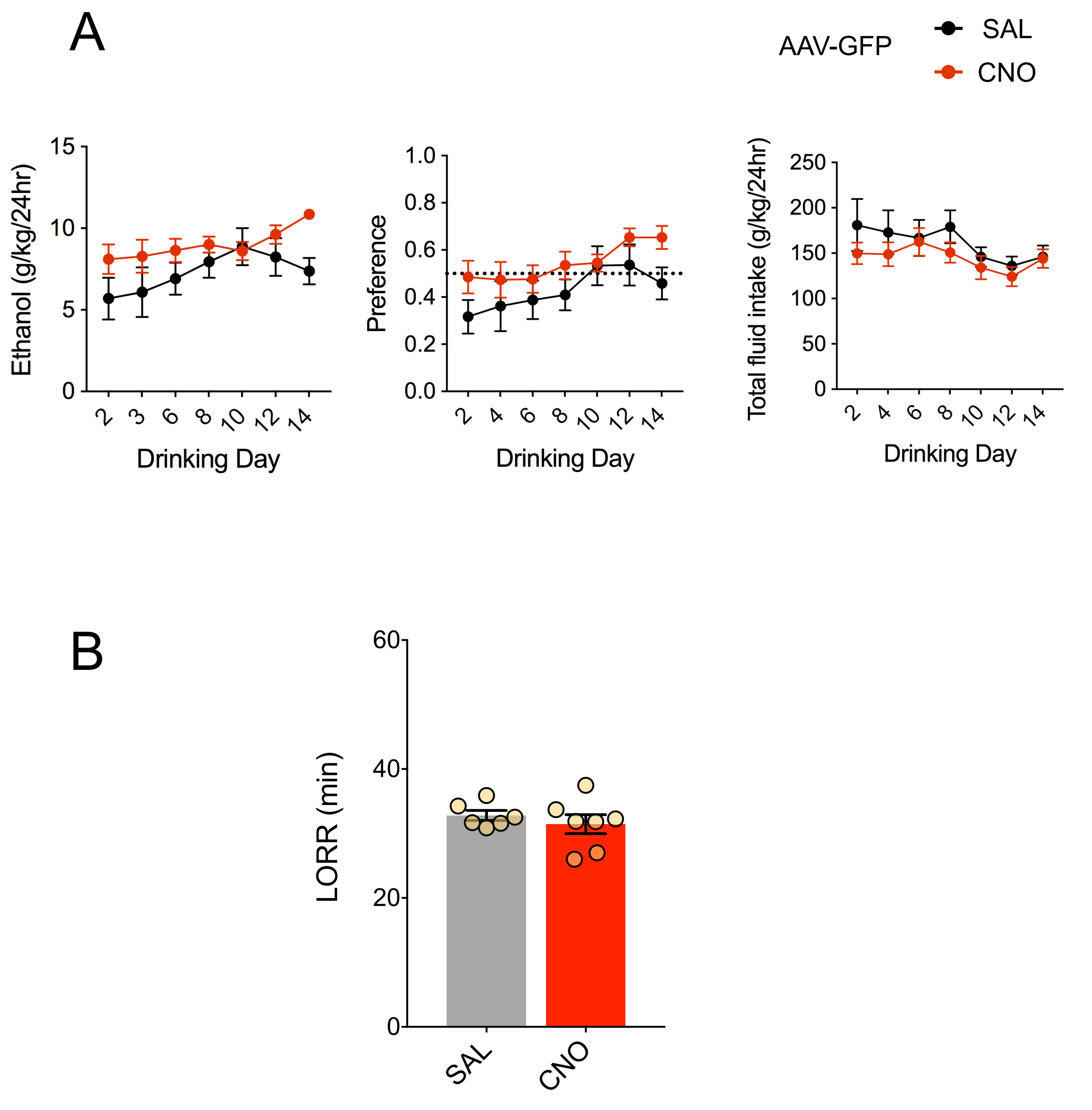

Supplementary Figure 3. CNO (1mg/kg) administered to non-DREADD-expressing mice did not affect ethanol consumption or ethanol-induced sedation. (**A**) AAV-GFP mice treated with CNO (red lines) do not exhibit altered ethanol drinking levels, ethanol preference, or total fluid consumption compared with saline-treated mice (black lines) (n = 6 per group). (**B**) Ethanol (3.6 g/kg)-induced LORR duration in CNO- vs. saline-pretreated non-DREADD-expressing mice (n = 6-7 per group). Values are mean ± SEM.

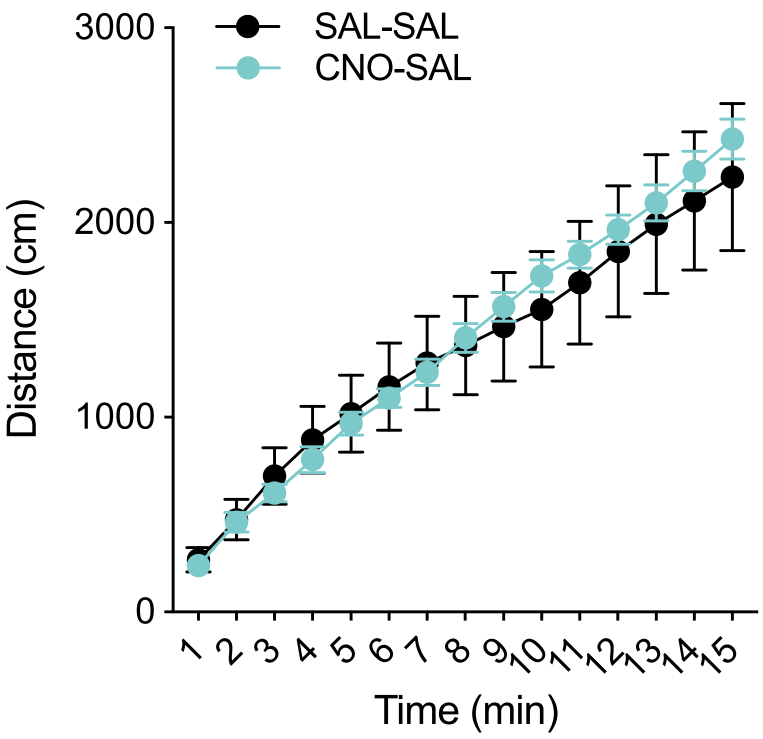

Supplementary Figure 4. Astrocyte G_q-DREADD activation did not change baseline locomotor activity in AAV-G_q-DREADD mice. Cumulative distance over a 15-minute period in saline- or CNO-pretreated AAV-G_q-DREADD-expressing mice treated with saline (SAL) 5 min before being placed in an activity-recording chamber (n = 6 per group). Values are mean ± SEM.

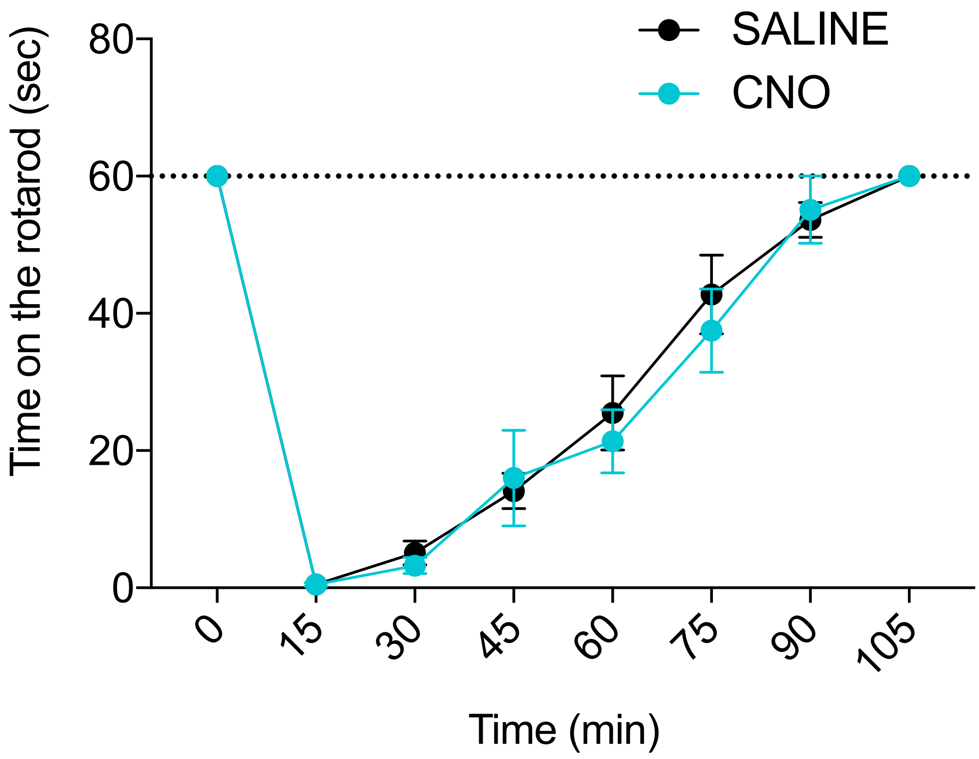

Supplementary Figure 5. Ethanol-induced motor incoordination is not affected by astrocyte activation. AAV-G_q-DREADD mice were trained to stay balanced on a rotating rod for 60 seconds. Next, mice were administered CNO (1mg/kg) or saline (i.p.) 30 minutes before an i.p. injection of 2g/kg ethanol. Mice were tested on their ability to stay balanced on the rotating rod. Latency to fall was recorded every 15 minutes until mice could stay on rotarod for 60 seconds (n = 8 per group). Values are mean ± SEM.
